# Gastroesophageal reflux-associated chronic cough in an adolescent and the diagnostic implications: a case report

**DOI:** 10.1186/1745-9974-4-5

**Published:** 2008-07-15

**Authors:** Makiko Jinnai, Akio Niimi, Masaya Takemura, Hisako Matsumoto, Yoshitaka Konda, Michiaki Mishima

**Affiliations:** 1 Department of Respiratory Medicine, Kyoto University, Shogoin, Sakyo-ku, Kyoto, 606-8507, Japan; 2 Department of Respiratory Medicine, The Tazuke Kofukai Medical Research Institute Kitano Hospital, 2-4-20 Ohgimachi, Kita-ku, Osaka, Japan; 3 Department of Internal Medicine, Japan Baptist Hospital, 47 Yamanomoto-cho, Kitashirakawa, Sakyo-ku, Kyoto, 606-8273, Japan

## Abstract

A 15-year-old girl was referred with a 2-year history of perennial non-productive cough, which had been preceded by *Mycoplasma pneumoniae *pneumonia and subsequent asthma. Symptoms were only partially responsive to anti-asthma treatment including an inhaled corticosteroid and a leukotriene receptor antagonist. The patient's BMI was 27.8; she had gained over 10 kg in the previous two years. Typical symptoms of gastroesophageal reflux disease were not evident except for belch. Coughing worsened on eating and rising from bed. Although esophagography failed to disclose reflux esophagitis, esophageal pH monitoring revealed significant acid reflux. Asthma was considered well controlled. Treatment with the proton-pump inhibitor rabeprazole resulted in disappearance of cough. Frequency Scale for the Symptoms of Gastroesophageal reflux disease (FSSG) score, a questionnaire evaluating the symptoms of gastroesophageal reflux disease, was initially high but normalized after treatment. Capsaicin cough sensitivity also diminished with treatment.

Chronic cough due to gastroesophageal reflux disease has been considered rare in adolescents, but this condition might be increasing in line with the recent trend in adults. Clinical features of gastroesophageal reflux disease-associated cough typical for adult patients and a specific questionnaire for evaluating gastroesophageal reflux disease validated in adults may also be useful diagnostic clues in adolescents.

## Background

Cough is the most common symptom for which patients seek medical attention. In adults, cough variant asthma, postnasal drip or rhinosinusitis, and gastroesophageal reflux disease (GERD) are the most common causes of chronic cough in Western countries[1]. In Japan, cough variant asthma, sinobronchial syndrome, and atopic cough have been considered the major causes of chronic cough lasting for 8 weeks or longer[2], but the prevalence of GERD is likely increasing [3-5], as has been reported in the USA[6]. There are far fewer studies of chronic cough etiology in children than in adults, but GER is considered rare, especially in adolescents [7-9].

We report a case of chronic cough due to GERD which presumably started at 13 years of age. Clinical features typical in adult patients[10] and a specific questionnaire for evaluating GERD validated in adults[11] were useful in leading us to suspect GER-related cough before considering esophagoscopy and esophageal pH monitoring.

## Case Presentation

**Figure 1:**
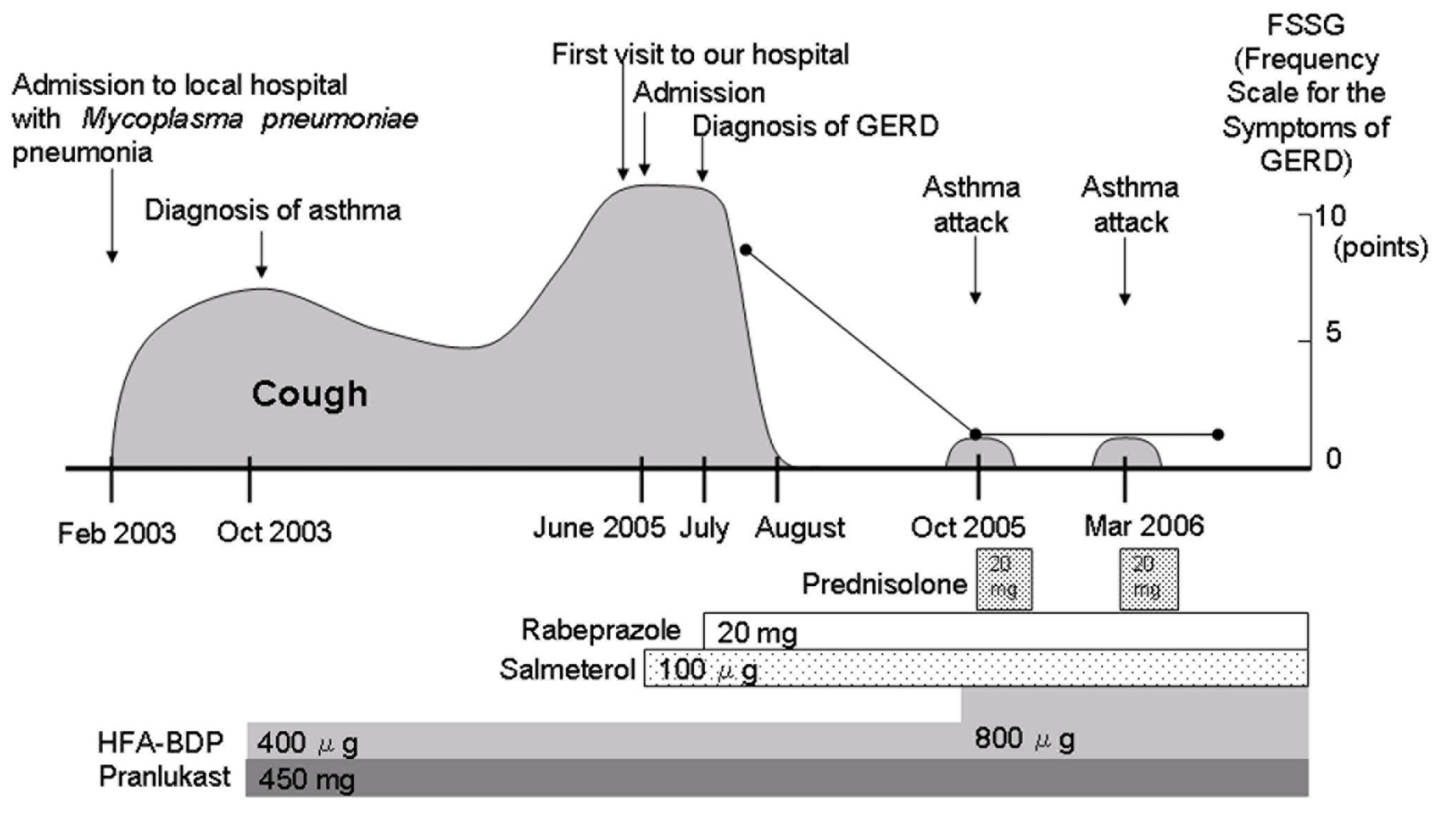
The patient's clinical course.

In February 2003, a 13-year-old girl was admitted to a local hospital because of fever, cough and chest infiltrate in X-ray. She was diagnosed as having *Mycoplasma pneumoniae *pneumonia from serology. Fever and chest infiltrate resolved rapidly with antibiotic treatment, but cough and wheezing persisted for several months. A diagnosis of asthma was made, and treatment with inhaled hydrofluoroalkane beclomethasone dipropionate 200 μg bid and an leukotriene receptor antagonist pranlukast 225 mg bid was started. Although wheezing resolved with this treatment, cough only partially improved and persisted. In May 2005 she was again admitted to hospital due to an exacerbation of coughing that prevented her attending school, but investigations including laryngoscopy and pulmonary function tests were normal. The patient was referred and admitted to our department in June 2005 (Figure 1).

The patient was afebrile and in good general condition. Her height and weight were 162 cm and 73 kg, respectively, with a BMI of 27.8. Physical examination including chest auscultation was normal, as were radiographs of the chest and sinus. Methacholine airway hyperresponsiveness was positive, but spirometry results were normal as indicated by an FVC of 3.8 L (120% of predicted value), an FEV_1 _of 3.31 L (120%), and an FEV_1_/FVC of 87%. Bronchial reversibility was negative as demonstrated by pre- and post-salbutamol FEV_1 _values of 3.31 L and 3.29 L, respectively. Peak expiratory flow ranged from 420 to 440 L/min (variation < 5%), and eosinophil count in induced sputum was normal (0.5%)[12]. Addition of inhaled salmeterol did not improve the patient's cough. These findings indicated that asthma was well controlled, and unlikely to be the cause of persistent cough. High resolution lung CT was unremarkable. Cough sensitivity to capsaicin was slightly heightened (C5, the lowest concentration of capsaicin required to induce 5 coughs, was 4.88 μM)[13].

The patient lacked typical esophageal symptoms of GERD such as heartburn or regurgitation, but complained of belch. She was obese with a weight gain of over 10 kg in the last two years. Cough was predominant in the daytime and deteriorated on rising from bed and after eating. Frequency Scale for the Symptoms of Gastroesophageal reflux disease (FSSG) score, a questionnaire evaluating the symptoms of GERD, was 9 points, which was higher than the reference value (8 points)[11]. GER was accordingly suspected as the cause of persistent cough. Esophagoscopy failed to disclose reflux esophagitis, but 24-hour esophageal pH monitoring revealed significant acid reflux: pH was below 4.0 for 17% of the whole examination period; this is 4 times higher than the reference value for children (4%)[14] and that for adults (4.2%)[15]. Treatment with rabeprazole, a proton-pump inhibitor, was started (20 mg daily), and the patient's cough was markedly relieved, eventually disappearing after 4 weeks of treatment. FSSG score decreased to 2 points after 3 months, and after one year C5 had also increased to 19.5 μM, indicating improved sensitivity to capsaicin.

The patient remains on treatment for asthma and GERD to date. In addition to continued use of rabeprazole, the patient has lost 10 kg by following a reducing diet. She has had several asthma exacerbations, but episodes have subsided with short courses of oral prednisolone. Otherwise coughing has been absent (Figure 1).

## Discussion

Three prospective studies by Irwin et al. over a period of 17 years have shown that GERD has increased in importance as the cause of chronic cough in adults[6]: 10% (the 4th commonest cause) in 1981; 21% (3rd) in 1990[1]; and 36% (2^nd^) in 1998. Chronic cough due to GERD was once considered rare in Japan[2], but among patients with chronic cough at our clinic, GERD has increased as the cause from 2% to around 10% over a decade [3-5] to become the 3^rd ^commonest cause[5].

Few studies have addressed the causes of chronic cough in children, but available results suggest that GERD is rare as a cause of isolated cough, especially in those aged 1 year or older[7-9,16]. Marchant et al.[17] recently reported that the prevalence of GERD in 108 children with cough (median age 2.6 years; duration > 3 weeks) was 3.0% but in none of the children was cough solely ascribed to GERD. Holinger studied 38 children (aged 3 months to 15 years) with cough (> 4 weeks) but found only one with GERD[7]. A later study by Holinger found GERD responsible for cough (>4 weeks) in 11 out of 72 infants and children[18]. In that study, although GERD was the most common cause of cough among infants aged 18 months or younger (9 of 32, 28%), it was the cause of cough in only one of 22 children aged 6 to 16 years[18]. GERD commonly occurs in infants[19] and becomes symptomatic during the first months of life, peaks by 4–5 months, and resolves by 12–24 months in most affected babies[20,21]. This may explain the fact that high prevalence of GERD-associated cough is limited to very young children[7,18]. The epidemiology of chronic cough in Japanese children is poorly known, but the prevalence of GERD may also have been low until recently. In a preliminary investigation, coughing was attributed to GERD in only 2 of 58 children (median age 5.2 years)[22]. However, the evidence in adults [3-6] leads us to suspect that GERD might be increasing as a cause of chronic cough, especially in older children or adolescents.

The golden standard for the diagnosis of GERD was 24-hr esophageal pH monitoring formerly, but has recently been taken place by multi-channel intraluminal impedance-pH monitoring that can detect non-acid reflux[23,24]. In any case, however, these examinations are invasive and not widely available. As clinical clues to the diagnosis of GERD, typical symptoms such as heartburn, regurgitation, and belch are important[9]. In a recent study, the commonest symptoms of 47 adult patients with chronic cough and objectively proven GER included cough on phonation, cough on rising from bed, cough on eating, and dysphonia[10]. Increased BMI has been associated with symptoms of GERD, and even moderate weight gain may cause or exacerbate symptoms of reflux[25]. These features reported in adults were helpful in raising the suspicion of GERD-related cough in our patient. Such information has been scarce for children, as gastroesophageal cough is considered rare in this age group[26].

FSSG score is a simplified questionnaire for evaluating the symptoms of GER, and it has been validated on the basis of endoscopic evidence of reflux esophagitis in Japanese adults[11]. When the cutoff score was set at 8 points, FSSG had a sensitivity of 62%, a specificity of 59%, and an accuracy of 60%[11]. Moreover, its responsiveness to intervention is high[11]. Our patient may be the first with GER-associated chronic cough to demonstrate a high FSSG score that responded well to treatment. The PPI was not ceased to see if the cough recurred in our patient, but we are confident that GERD was responsible for the patient's longstanding cough that was quickly relieved by the PPI.

In our patient, cough was attributed to asthma before the diagnosis of GERD was established. Chronic cough often has dual causes, and GERD is an important consideration because a self-perpetuating positive feedback cycle between cough and GER has been demonstrated[27,28]. Cough from any cause may precipitate further reflux, leading to a vicious cycle of cough persistence[27,28]. When cough improves only partially with conventional treatment of the primary diagnosis, coexistence of GERD needs to be considered.

## List of abbreviations

GERD: Gastroesophageal reflux; FSSG: Frequency scale for symptoms of gastroesophageal reflux disease.

## Competing interests

The authors declare that they have no competing interests.

## Authors' contributions

MJ carried out the pulmonary function and methacholine challenge tests and wrote the initial draft of the manuscript. AN was responsible for disease diagnosis and management, revision of the manuscript, and supervision of the study. MT carried out the capsaicin challenge test and was also responsible for disease diagnosis and management. HM participated in disease management. YK performed the esophageal pH monitoring and interpreted the results. MM supervised the study. All authors read and approved the final manuscript.

## Consent

Written informed consent was obtained from the patient for publication of this case report and any accompanying images. A copy of the written consent is available for review by the Editor-in-Chief of this journal.
